# Metabolic health in Bangladesh: trends and challenges

**DOI:** 10.1186/s43162-022-00166-0

**Published:** 2022-10-22

**Authors:** Saifur Rahman Chowdhury

**Affiliations:** 1 Department of Health Research Methods, Evidence, and Impact, McMaster University, Hamilton, ON Canada; 2 Department of Public Health, North South University, Dhaka, Bangladesh

To the Editor,

Bangladesh is a lower-middle-income country located in the South-East Asian region and has a population of over 170 million. The country faces many challenges in terms of health inequality and preventative strategies, in particular for noncommunicable diseases. Over the course of the previous half-century, Bangladesh has experienced a reduction in the prevalence of infectious diseases and an increase in the prevalence of chronic diseases [1]. The socioeconomic and cultural transition of Bangladesh has contributed to significant shifts in the routine lifestyle and dietary practices of the country’s people, which has resulted in an increase in the prevalence of metabolic disorders.

A nationwide cross-sectional study conducted in 2018 reported that the prevalence of insufficient physical activity among Bangladeshi adults was 12.3%, whereas the women (14.8%) and urban (14.1%) populations showed the greater frequency [2]. The study also revealed that the prevalence of overweight and obesity was 25.9% and was significantly higher in the women (33.7%), urban (34.3%), and richest (34.3%) populations [2]. These results are clearly indicating an increasing trend of anthropometric measurements compared to the findings of a study conducted in 2010 (18% was overweight) [3].

As a consequence, the statistics of metabolic syndrome, a cluster of metabolic and cardiovascular abnormalities, are drastic for Bangladesh. For example, a meta-analysis that included the studies up to 2017 revealed that the prevalence of metabolic syndrome was 30.0%, and the prevalence of high fasting glucose was 28% [4]. Alarmingly, the prevalence of low HDL cholesterol was estimated at 89%. Similarly, the prevalence of high triglycerides was reported as high as 26%. The authors performed meta-regression, and a significantly higher weighted pooled prevalence (37% versus 14%) of metabolic syndrome was observed in the studies conducted in the year 2012 and afterward. The authors estimated that the overall prevalence of metabolic syndrome increases by 3.68% for every 1 year increase in the time of the study conducted [4].

The growing prevalence of type 2 diabetes in Bangladesh is also concerning. A recent nationwide survey reported that the prevalence of diabetes was 9.2% and was significantly higher in the urban (11.8%), elderly (14.8%), and richest (16.5%) populations [5]. A meta-analysis also showed a consistent result that the pooled prevalence of diabetes in the general population was 7.8%. The highest prevalence was found in people aged 50 and up, and the overall prevalence increased with age. The prevalence of diabetes is stratified by publication periods: 1995–2000, 2001–2010, and 2011–2019, and it was 4.0%, 6.3%, and 10.4%, respectively [6].

The coronavirus disease 2019 (COVID-19) pandemic is consuming huge public health resources worldwide and has exacerbated existing health inequalities though the countries have taken preventive strategies [7]. Moreover, evidence suggests that hypertension, cardiovascular disease, and diabetes are recognized as influential factors that contribute to poorer outcomes of COVID-19 [8].

Metabolic diseases lead to fatal consequences for COVID-19 and other communicable and noncommunicable diseases. Bangladesh is currently dealing with the metabolic repercussions of a recent shift in epidemiology. In order to combat the negative effects of metabolic disorders and reverse this trend, it is necessary for healthcare and civil society organizations to work together, in addition to the vigorous government efforts [1]. It is imperative that educational programs be implemented as quickly as possible and improvements be made to public facilities in order to alleviate the burden and fatal effects of poor metabolic health to assist Bangladeshis in having a healthy lifestyle.

## Data Availability

Not applicable.

## References

[1] Chowdhury SR (2022). Cardiovascular health and COVID-19: a Bangladesh perspective. Public Health Toxicol.

[2] Riaz BK, Islam MZ, Islam ANMS, Zaman MM, Hossain MA, Rahman MM (2020). Risk factors for non-communicable diseases in Bangladesh: findings of the population-based cross-sectional national survey 2018. BMJ Open.

[3] Bangladesh Society of Medicine, Directorate General of Health Services, World Health Organization, and Ministry of Health and Family Welfare B. Non-communicable disease risk factor survey Bangladesh 2010. 2011. Available: https://www.who.int/docs/default-source/searo/bangladesh/pdf-reports/year-2007-2012/non-communicable-disease-risk-factor-survey-bangladesh-2010.pdf?sfvrsn=37e45e81_2

[4] Ziaul M, Chowdhury I, Anik AM, Farhana Z, Bristi PD, Mamun BMA al et al (2018) Prevalence of metabolic syndrome in Bangladesh : a systematic review and meta- analysis of the studies. BMC Public Health. 18:1–14. 10.1186/s12889-018-5209-z 10.1186/s12889-018-5209-z PMC5833131 29499672

[5] Hossain MB, Khan MN, Oldroyd JC, Rana J, Magliago DJ, Chowdhury EK (2022). Prevalence of, and risk factors for, diabetes and prediabetes in Bangladesh: evidence from the national survey using a multilevel Poisson regression model with a robust variance. Sarker AR, editor. PLOS Global Public Health..

[6] Akhtar S, Nasir JA, Sarwar A, Nasr N, Javed A, Majeed R (2020). Prevalence of diabetes and pre-diabetes in Bangladesh: a systematic review and meta-analysis. BMJ Open..

[7] Chowdhury SR, Sunna TC, Sanjoy S (2020). Response to COVID-19 in Bangladesh: strategies to resist the growing trend of COVID-19 in a less restricted situation. Asia Pac J Public Health.

[8] Khan MMA, Khan MN, Mustagir G, Rana J, Islam MS, Kabir MI (2020). Effects of underlying morbidities on the occurrence of deaths in COVID-19 patients: a systematic review and meta-analysis. J Glob Health.

